# Tumeur de la voie excrétrice supérieure et hydronéphrose due à un syndrome de la jonction pyélo-urétérale: une association rare

**DOI:** 10.11604/pamj.2018.31.201.16181

**Published:** 2018-11-22

**Authors:** Soufiane Ennaciri, Jean Paul Omana

**Affiliations:** 1 Service d'Urologie, Centre Hospitalier Universitaire Hassan II, Fès, Maroc

**Keywords:** Tumeur, voie excrétrice supérieure, hydronéphrose, jonction pyélo-urétéral, Tumor, upper urinary tract, hydronephrosis, pyelo-ureteral junction

## Abstract

The association between tumor of the urinary tract and hydronephrosis caused by a syndrome of the pyelo-ureteral junction is rare. Indeed, tumors of the upper urinary tract and hydronephrosis have generally a cause-effect relationship. This last is due, more often, to an obstruction caused by a tumor of the ureter or of the pyelo-ureteral junction. We report the case of a 66-year old patient with a history of smoking and right pyelonephritis, presenting with right lumbar pain intermittently evolving over several months without haematuria. Ultrasound showed a dilation of the pyelocaliceal cavities with major reduction of the corticomedullary index of the right kidney. Uroscan was in favor of cystic dysplasia of the right kidney as well as of right hydronephrosis associated with syndrome of pyelo-ureteral junction with budding intrarenal images leading to the suspicion of pyelic tumor. The assessment was completed by urinary cytology which was positive. Right laparoscopic nephroureterectomy was performed and pathologic examination confirmed the diagnosis of urothelial carcinoma of the upper urinary tract.

## Image en médecine

L'association tumeur de la voie excrétrice supérieure et hydronéphrose due à un syndrome de la jonction pyélo-urétérale est une entité rare. En effet, les tumeurs de la voie excrétrice supérieure et l'hydronéphrose ont classiquement une relation de cause à effet. Cette dernière est due le plus souvent à l'obstruction par une tumeur de l'uretère ou de la jonction pyélo-urétérale. Nous rapportons le cas d'un patient de 66 ans, aux antécédents de tabagisme et de pyélonéphrite droite, présentant des douleurs lombaires droites évoluant par intermittence depuis plusieurs mois sans hématurie. L'échographie a montré une dilatation des cavités pyélo-caliciels avec réduction majeur de l'index cortico-médullaire du rein droit. L'uroscanner a douté entre une dysplasie kystique du rein droit, et une hydronéphrose droite sur syndrome de la jonction pyélo-urétérale avec des images bourgeonnantes intra rénales faisant suspecter une tumeur pyélique. Le bilan a été complété par une cytologie urinaire quie est revenue positive. Une néphro-urétérectomie droite par laparotomie a été réalisé et l'examen anatomopathologique a confirmé qu'il s'agit bien d'un carcinome urothélial des voies excrétrices supérieures.

**Figure 1:**
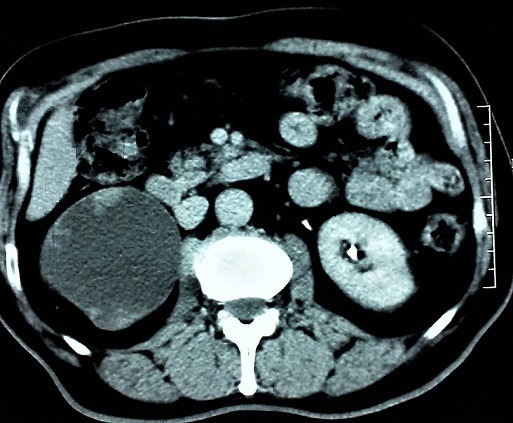
Uroscanner montrant une nette hydronéphrose droite avec des lésions bourgeonnantes intra pyélique faisant suspecter une tumeur de la voie excrétrice supérieure

